# Supplementary figures and images for: The Genetic Evolution of DENV2 in the French Territories of the Americas: A Retrospective Study from the 2000s to the 2024 Epidemic, Including a Comparison of Amino Acid Changes with Vaccine Strains
Source: Vaccines (Basel). 2025 Mar 1;13(3):264. doi: 10.3390/vaccines13030264 (PMC11945534; doi:10.3390/vaccines13030264)

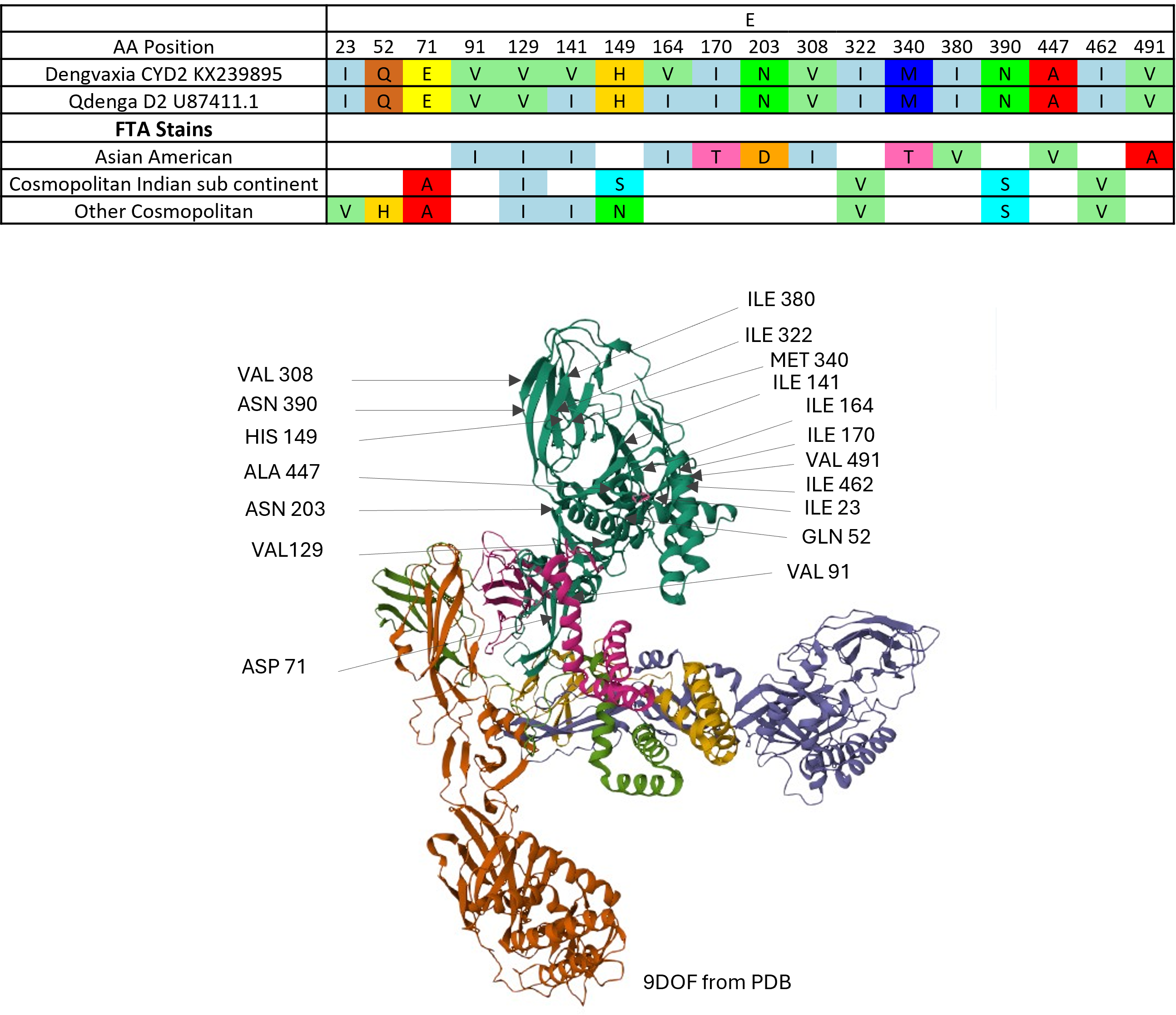

Supplement: Supplementary file 1 [file vaccines-13-00264-s001.zip › vaccines-3398813-supplementary.tif]
